# MeCorS: Metagenome-enabled error correction of single cell sequencing reads

**DOI:** 10.1093/bioinformatics/btw144

**Published:** 2016-03-15

**Authors:** Andreas Bremges, Esther Singer, Tanja Woyke, Alexander Sczyrba

**Affiliations:** ^1^Center for Biotechnology and Faculty of Technology, Bielefeld University, Bielefeld 33615, Germany; ^2^U.S. Department of Energy Joint Genome Institute, Walnut Creek, CA 94598, USA

## Abstract

**Summary:** We present a new tool, MeCorS, to correct chimeric reads and sequencing errors in Illumina data generated from single amplified genomes (SAGs). It uses sequence information derived from accompanying metagenome sequencing to accurately correct errors in SAG reads, even from ultra-low coverage regions. In evaluations on real data, we show that MeCorS outperforms BayesHammer, the most widely used state-of-the-art approach. MeCorS performs particularly well in correcting chimeric reads, which greatly improves both accuracy and contiguity of *de novo* SAG assemblies.

**Availability and implementation**: https://github.com/metagenomics/MeCorS

**Contact:** abremges@cebitec.uni-bielefeld.de

**Supplementary information:** Supplementary data are available at *Bioinformatics* online.

## 1 Introduction

The vast majority of microbial species found in nature has yet to be grown in pure culture, turning metagenomics and—more recently—single cell genomics into indispensable methods to access the genetic makeup of microbial dark matter (Brown *et al.*, 2015; Rinke *et al.*, 2013). Frequently, single amplified genomes (SAGs) and shotgun metagenomes are generated from the same environmental sample, and are methodologically combined e.g. to validate metagenome bins with single cells or to improve the SAG’s assembly contiguity (Campbell *et al.*, 2013; Hess *et al.*, 2011). However, a single cell’s DNA needs to be amplified prior to sequencing, as usually accomplished by multiple displacement amplification (MDA; Lasken, 2007). This amplification is heavily biased, leading to uneven sequencing depth including ultra-low coverage regions with basically no informed error correction possible (Chitsaz *et al.*, 2011; Supplementary Fig. S1). Moreover, chimera formation occurs roughly once per 10 kbp during MDA, further complicating SAG assembly (Nurk *et al.*, 2013; Rodrigue *et al.*, 2009).

While an array of error correction tools exist for a variety of use cases (Laehnemann *et al.*, 2016), only one tool was specifically designed to correct SAG data: hammer (Medvedev *et al.*, 2011), recently refined to BayesHammer (Nikolenko *et al.*, 2013). We propose a metagenome-enabled error correction strategy for single cell sequencing reads. Our method takes advantage of largely unbiased metagenomic coverage, enabling it to correct positions with too low a coverage for SAG-only error correction, and to correct chimeric SAG reads through non-chimeric metagenome reads.

## 2 Methods

We correct potential errors using an algorithm similar to solving the *spectral alignment problem* (Pevzner *et al.*, 2001). Given a set of trusted *k*-mers, we use a heuristic method to find a sequence with minimal corrections such that each *k*-mer on the corrected sequence is trusted. Using a *k*-mer size of 31, we consider a *k*-mer trusted if it occurs at least twice in the accompanying metagenome. This coverage threshold was determined empirically to work with most datasets (Supplementary Fig. S2).

Our correction algorithm was inspired by fermi (Li, 2012) and BFC (Li, 2015), but we do not act on the assumption of uniform sequencing coverage, thereby accounting for the tremendous variation of coverage across the SAG. Instead, we exploit metagenomic sequence information to correct errors resulting from amplification and sequencing, as well as chimeras, even in ultra-low coverage regions of the SAG. The non-chimeric nature of the metagenome reads enables an implicit and thorough write-through correction of chimeric SAG reads.

MeCorS works in three phases:

1. MeCorS collects all 31-mers (and their reverse complements) occurring in the SAG reads. It uses this information to initialize a hash table with the 31-mers being valid keys.
2. MeCorS scans the accompanying metagenomic reads. For each stored 31-mer, it counts the occurrence of the next (i.e. the 32nd) base in the metagenome and stores the totals in the hash table. This step is largely I/O bound and dominates MeCorS’s runtime.
3. MeCorS processes each SAG read by using the 31-mer hash table to check if the 32nd base is sufficiently supported in the metagenome. Untrusted 32nd bases are replaced with the most frequent and trusted 32nd bases from the metagenome.

## 3 Results and discussion

As a realistic benchmark, we used eight *Escherichia coli* K12-MG1655 SAGs from Clingenpeel *et al.* (2014), a strain for which the complete genome sequence is available (Supplementary Table S1). A concomitant *in vitro* mock metagenome consisting of 26 microbial species, including *E.* *coli* K12-MG1655, was sequenced on Illumina’s HiSeq platform (Bowers *et al.*, 2015). Based on metagenome read mapping, we estimate the relative abundance of *E. coli* to amount to 0.15%, corresponding to a mean per-base coverage of only 20.7× (Supplementary Table S2).

**Fig. 1.**
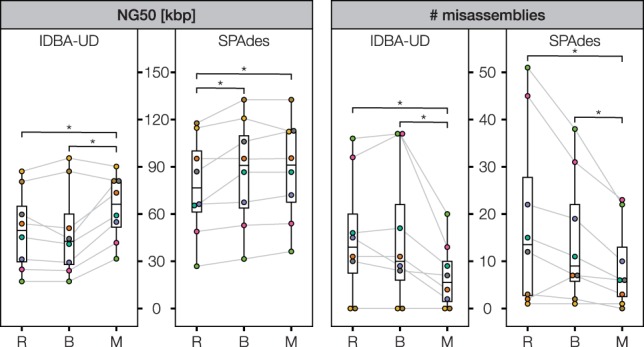
Effect on SAG assembly. We corrected the raw reads (R) with BayesHammer (B; Nikolenko *et al*., 2013) or MeCorS (M). We then used IDBA-UD (Peng *et al*., 2012) and SPAdes (Bankevich *et al*., 2012) to assemble the SAGs. Brackets indicate all statistically significant changes (P < 0.05; two-tailed Wilcoxon signed-rank test). Quality assessment with QUAST (Gurevich *et al*., 2013); Supplementary Tables S4 and S5 contain in-depth assembly statistics

We evaluated MeCorS along with BayesHammer (Nikolenko *et al.*, 2013), a widely used error correction tool for SAG data. Our method corrects more errors than BayesHammer, producing a significantly higher fraction of better and perfect reads after correction (Table 1; Supplementary Table S3). In contrast to BayesHammer, MeCorS reduces the amount of chimeric SAG reads by one order of magnitude, likely due to the non-chimeric nature of the metagenome reads. MeCorS works well with modern single cell assemblers, most notably reducing the misassembly rate of both IDBA-UD (Peng *et al.*, 2012) and SPAdes (Bankevich *et al.*, 2012) by half, while providing high sequence contiguity (Fig. 1). In particular poorly amplified SAGs benefit from metagenome-enabled error correction, yielding improved assembly accuracy and contiguity (Supplementary Tables S4 and S5).

**Table 1.** Performance of SAG error correction

Program	% perfect	% chimeric	% better	% worse
Raw	22.52 ± 1.07	0.73 ± 0.15	–	–
BayesHammer	80.35 ± 8.77	0.77 ± 0.17	71.66 ± 2.12	0.33 ± 0.06
MeCorS	95.52 ± 0.43	0.06 ± 0.02	75.45 ± 1.11	0.26 ± 0.03

Mean percentage and standard deviation of *perfect* reads, *chimeric* reads (i.e. reads with parts mapped to different places), corrected reads becoming *better* and *worse* than the raw reads. Evaluation as described in Li (2015); please refer to Supplementary Table S3 for per-SAG metrics, including runtime and memory usage.

We note that such a hybrid error correction of SAG data may result in miscorrection(s) of rare variants. If the captured cell contains a variant that is rare or absent in the corresponding metagenome, correction will be biased towards the most abundant variant in the metagenome sequence. If strain resolution is desired, we suggest polishing the SAG assembly using the uncorrected raw data. In all other cases, SAG assemblies benefit directly from metagenome-enabled error correction via MeCorS.

Uneven genome coverage and chimera formation present the biggest challenges in the downstream processing and analysis of SAG datasets to date. We propose MeCorS for the correction of SAG reads when complementary metagenome datasets are available. Error and chimera correction is essential for improved SAG assembly and demonstrates a powerful application of combined shotgun metagenome and single cell sequencing.

## Funding

A.B. is supported by a fellowship from the CLIB Graduate Cluster Industrial Biotechnology and is partially funded by the International DFG Research Training Group GRK 1906/1. The work conducted by the U.S. Department of Energy Joint Genome Institute, a DOE Office of Science User Facility, is supported under Contract No. DE-AC02-05CH11231.

*Conflict of Interest*: none declared.

## Supplementary Material

Supplementary Data
