# Supplementary material for: MeCorS: Metagenome-enabled error correction of single cell sequencing reads
Source: Bioinformatics. 2016 Mar 15;32(14):2199–201. doi: 10.1093/bioinformatics/btw144 (PMC4937190; doi:10.1093/bioinformatics/btw144)
Supplement: Supplementary Data [file supp_32_14_2199__index.html]

MeCorS: Metagenome-enabled error correction of single cell sequencing reads — MeCorS: Metagenome-enabled error correction of single cell sequencing reads — Supplementary Data 

# MeCorS: Metagenome-enabled error correction of single cell sequencing reads

## Supplementary Data

files

- Supplementary Data - pdf file
