# Supplementary material for: MeCorS: Metagenome-enabled error correction of single cell sequencing reads
Source: Bioinformatics. 2016 Mar 15;32(14):2199–201. doi: 10.1093/bioinformatics/btw144 (PMC4937190; doi:10.1093/bioinformatics/btw144)
Supplement: Supplementary Data [file supp_btw144_MeCorS_Supplement.pdf]

Supplementary Information

Bremges, A.[1], Singer, E., Woyke, T. and Sczyrba, A. (2016). MeCorS: Metagenome-enabled error correction of single cell sequencing reads.

[1] abremges@cebitec.uni-bielefeld.de

Availability

MeCorS git repository	https://github.com/metagenomics/MeCorS
Benchmarking data sets	http://genome.jgi.doe.gov/MeCorS

List of Figures

S1 SAG sequencing coverage profiles 2
S2 Effect of different coverage thresholds 6
S3 Effeciency of threading 9

List of Tables

S1 Profile of the *in vitro* mock metagenome 3
S2 Performance of SAG error correction 4
S3 IDBA-UD assembly results 7
S4 SPAdes assembly results 8

E. coli single amplified genomes

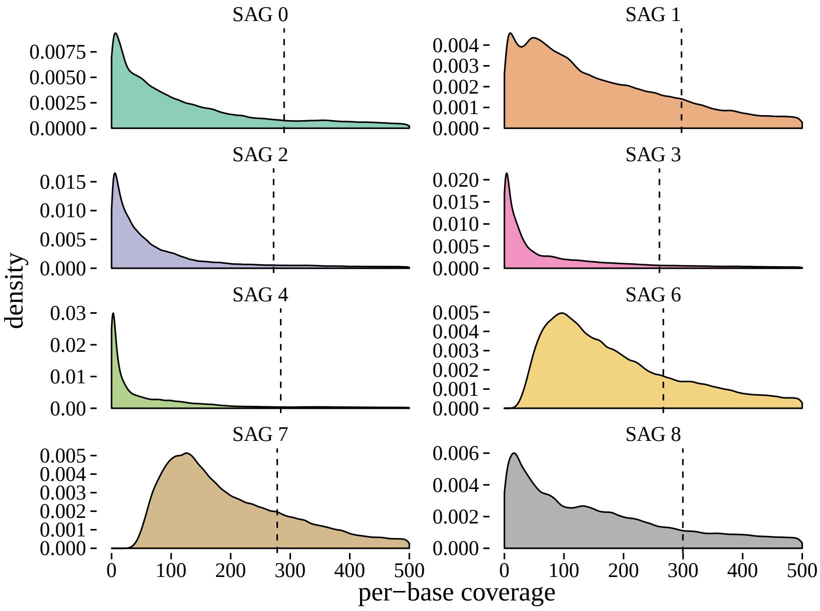

Figure S1: **SAG sequencing coverage profiles.** Density estimations of the per-base coverages, dotted lines represent the SAG's mean coverage.

For benchmarking, we used eight *Escherichia coli* K12-MG1655 single amplified genomes (SAGs) from Clingenpeel *et al.*, 2014[2]:

> "Single-cell genomes were obtained as described in Clingenpeel *et al.* (2014)[3]. Reads were subsampled to 315x per genome. Reads were filtered for human contamination by alignment using bwa (Li and Durbin, 2010)[4] and for Illumina artifacts using an in-house tool duk (unpublished)."

[2] Clingenpeel, S. *et al.* (2014b). Reconstructing each cell's genome within complex microbial communities–dream or reality? *Front Microbiol*, **5**, 771

[3] Clingenpeel, S. *et al.* (2014a). Effects of sample treatments on genome recovery via single-cell genomics. *ISME J*, **8**(12), 2546–2549

[4] Li, H. and Durbin, R. (2010). Fast and accurate long-read alignment with Burrows-Wheeler transform. *Bioinformatics*, **26**(5), 589–595

While the mean per-base coverage is similar across SAGs, coverage bias introduced by multiple displacement amplification (MDA) results in quite variable profiles (Figure S1). Two SAGs, 6 and 7, show hardly any bias and resemble isolate-grade genomes with a Poisson-like coverage distribution. All other SAGs suffer from considerabe MDA bias.

The interquartile range (IQR) of the per-base coverage indicates the variation of coverage across the SAG. Based on the IQR, we propose a "quality" ranking of the eight *E. coli* SAGs:

$$6 \approx 7 > 1 \approx 8 > 0 > 2 > 3 > 4$$

Poorly amplified SAGs benefit the most from error correction with MeCorS (Table S2). The quality of a SAG assembly seems to be negatively correlated with its coverage variation (Tables S3 and S4).

In vitro mock metagenome

Taxonomy ID	Phylum	Species	mapped reads	avg. coverage	est. abundance
771875	Thermotogae	*Fervidobacterium pennivorans*	39566833	2708.24	19.39%
646529	Firmicutes	*Desulfosporosinus acidophilus*	53202915	1579.64	11.31%
526227	Deinococcus-Thermus	*Meiothermus silvanus*	32231620	1276.02	9.14%
573413	Spirochaetes	*Spirochaeta smaragdinae*	39431130	1255.97	8.99%
582402	Proteobacteria	*Hirschia baltica*	28144226	1181.16	8.46%
717605	Firmicutes	*Thermobacillus composti*	30954326	1046.39	7.49%
767817	Firmicutes	*Desulfotomaculum gibsoniae*	24329020	741.83	5.31%
767434	Proteobacteria	*Frateuria aurantia*	13922996	568.16	4.07%
633147	Actinobacteria	*Olsenella uli*	7839526	552.99	3.96%
768704	Firmicutes	*Desulfosporosinus meridiei*	16066750	487.57	3.49%
583355	Verrucomicrobia	*Coraliomargarita akajimensis*	11810956	467.03	3.34%
694430	Euryarchaeota	*Natronococcus occultus*	12505736	403.70	2.89%
797304	Euryarchaeota	*Natronobacterium gregoryi*	8676937	335.23	2.40%
797302	Euryarchaeota	*Halovivax ruber*	6060380	274.95	1.97%
926566	Acidobacteria	*Terriglobus roseus*	8464573	239.33	1.71%
640132	Actinobacteria	*Segniliparus rotundus*	4886507	225.63	1.62%
644801	Proteobacteria	*Pseudomonas stutzeri*	5448168	174.50	1.25%
160490	Firmicutes	*Streptococcus pyogenes*	1502208	120.44	0.86%
195103	Firmicutes	*Clostridium perfringens*	1461840	66.55	0.48%
203119	Firmicutes	*Clostridium thermocellum*	1542460	59.62	0.43%
882884	Proteobacteria	*Salmonella enterica*	1831145	58.72	0.42%
926556	Bacteroidetes	*Echinicola vietnamensis*	2160987	57.22	0.41%
196627	Actinobacteria	*Corynebacterium glutamicum*	1063668	47.67	0.34%
**511145**	**Proteobacteria**	***Escherichia coli***	**647555**	**20.65**	**0.15%**
218493	Proteobacteria	*Salmonella bongori*	501312	16.61	0.12%
446468	Actinobacteria	*Nocardiopsis dassonvillei*	6640	0.06	$< 0.01\%$

Table S1: **Profile of the *in vitro* mock metagenome.** 26 microbial species.

For benchmarking, we used the *in vitro* mock metagenome, including *E. coli* K12-MG1655, from Bowers *et al.*, 2015[5]:

> "The mock community is composed of 23 bacterial species and 3 archaeal species. DNA from pure cultures of each of the 26 microbial taxa was extracted with standard genomic purification kits. DNA extracts were quantified in quadruplicate with the Qubit 2.0 fluorometer and pooled at varying ratios to produce a mock community [...] The unamplified control library was prepared from a 200 ng aliquot of the pooled mock community DNA using Illumina's TruSeq library preparation protocol. [...] All libraries were sequenced on the Illumina HiSeq 2000 platform using 2 x 150 bp paired end sequencing."

[5] Bowers, R. M. *et al.* (2015). Impact of library preparation protocols and template quantity on the metagenomic reconstruction of a mock microbial community. *BMC Genomics*, **16**(1), 856

Shotgun metagenome sequencing generated a total of 355,875,608 reads (53 Gbp). We mapped these with BWA-MEM[6], version 0.7.12, simultaneously against all 26 reference genomes, postprocessed the alignments with samtools[7], version 1.2, and calculated the per-base coverage values (Table S1).

The relatively low abundance of *E. coli* in the mock community (0.15%) results in a mean coverage of 20.7x, indicating that MeCorS can correct chimeric reads and sequencing errors even when facing only minimal metagenomic coverage (Table S2).

[6] Li, H. (2013). Aligning sequence reads, clone sequences and assembly contigs with BWA-MEM. *arXiv:1303.3997*

[7] Li, H. *et al.* (2009). The Sequence Alignment/Map format and SAMtools. *Bioinformatics*, **25**(16), 2078–2079

SAG error correction

Metric	Program	SAG							
		0	1	2	3	4	6	7	8
Reads	–	9365134	9604918	8811278	8396488	9257066	8609900	8990744	9682468
Perfect	raw	2120932	2179609	1937541	1954244	1872800	2049675	2063874	2183454
	BayesHammer	7656274	8260510	6302861	5970186	6297298	7639715	8068555	8317006
	MeCorS	**8886436**	**9188854**	**8440502**	**7965810**	**8829995**	**8264229**	**8611867**	**9272559**
Chimeric	raw	69568	67625	81938	75509	79443	52246	44983	59265
	BayesHammer	72820	70590	87564	80336	84813	53875	46387	61948
	MeCorS	**5502**	**4593**	**10397**	**4648**	**5257**	**3941**	**3824**	**4889**
Better	raw	–	–	–	–	–	–	–	–
	BayesHammer	6743983	7026478	6156387	5669978	6574627	6244274	6645542	7095951
	MeCorS	**7008163**	**7236195**	**6707136**	**6260408**	**7206731**	**6403639**	**6749255**	**7306961**
Worse	raw	–	–	–	–	–	–	–	–
	BayesHammer	31644	26951	32315	29096	41584	25270	26959	28960
	MeCorS	**25990**	**25304**	**20560**	**27335**	**27390**	**20791**	**21074**	**24001**
Time (h)	BayesHammer	1:43	1:45	1:51	2:04	2:24	1:35	1:38	1:46
	MeCorS	**1:13**	**1:09**	**0:58**	**0:53**	**1:06**	**1:00**	**1:08**	**1:13**
RAM (GB)	BayesHammer	14.90	15.64	13.70	12.34	15.33	14.59	15.72	15.77
	MeCorS	**10.77**	**10.75**	**10.76**	**9.65**	**10.76**	**10.79**	**10.75**	**10.75**

Table S2: **Performance of SAG error correction.** Time/RAM for 16 threads.

We corrected the raw reads with BayesHammer[8], bundled with SPAdes[9], version 3.6.0. We disabled quality trimming in SPAdes's `configs/hammer/config.info.template` by zeroing `input_trim_quality` to perform only error correction.

[8] Nikolenko, S. I. *et al.* (2013). BayesHammer: Bayesian clustering for error correction in single-cell sequencing. *BMC Genomics*, **14 Suppl 1**, S7

[9] Bankevich, A. *et al.* (2012). SPAdes: a new genome assembly algorithm and its applications to single-cell sequencing. *J. Comput. Biol.*, **19**(5), 455–477

We ran BayesHammer using SPAdes's wrapper script:

```
for f in Eco*.fastq.1.gz; do
	spades.py --sc --only-error-correction -1 $f -2 ${f/.1./.2.} \
	-o hammer_${f%.fastq.1.gz}
done
```

We also corrected the raw sequencing reads with MeCors:

```
MG=/path/to/metagenome.fastq
for f in Eco*.fastq.?.gz; do
	mecors -s $f -m $MG | gzip -1 > ${f/.fastq./.mecors.fastq.}
done
```

We evaluated the performance of read error correction as described in Li, 2015[10], using the same read-based metrics:

[10] Li, H. (2015). BFC: correcting Illumina sequencing errors. *Bioinformatics*, **31**(17), 2885–2887

> "A read is said to become *better* (or *worse*) if the best alignment of the corrected sequence has more (or fewer) identical bases to the reference genome than the best alignment of the original sequence. The table gives [. . .] the number of reads mapped *perfectly*, number of *chimeric* reads (i.e. reads with parts mapped to different places), number of corrected reads becoming *better* and the number of corrected reads becoming *worse* than the original reads."

We note that MeCorS only implicitely corrects chimeric SAG reads, yet reduces the amount of chimeras by one order of magnitude. MDA introduces chimeric junctions roughly once per 10 kbp in SAGs[11], but metagenome sequencing is largely unbiased and free of chimeras.

[11] Lasken, R. S. and Stockwell, T. B. (2007). Mechanism of chimera formation during the Multiple Displacement Amplification reaction. *BMC Biotechnol.*, 7, 19

Chimeric reads contain DNA sequences originating from two different genome regions, say A and B, with the first part originating from region A, the second part from region B. A chimeric junction will (in most cases) result in an untrusted 32nd base (from region B) when looking at its 31-mer prefix (from region A; phase 3 of MeCorS). MeCorS then tries to correct this position of the SAG read by replacing the untrusted 32nd base (B) with the most frequent and trusted 32nd base from the metagenome (A). MeCorS therefore performs an implicit and thorough write-through correction of chimeric SAG reads, completely rewriting their second parts.

Fine-tuning MeCorS

We recommend running MeCorS with default settings. They work sufficiently well for most SAG/metagenome combinations.

Metagenome coverage threshold

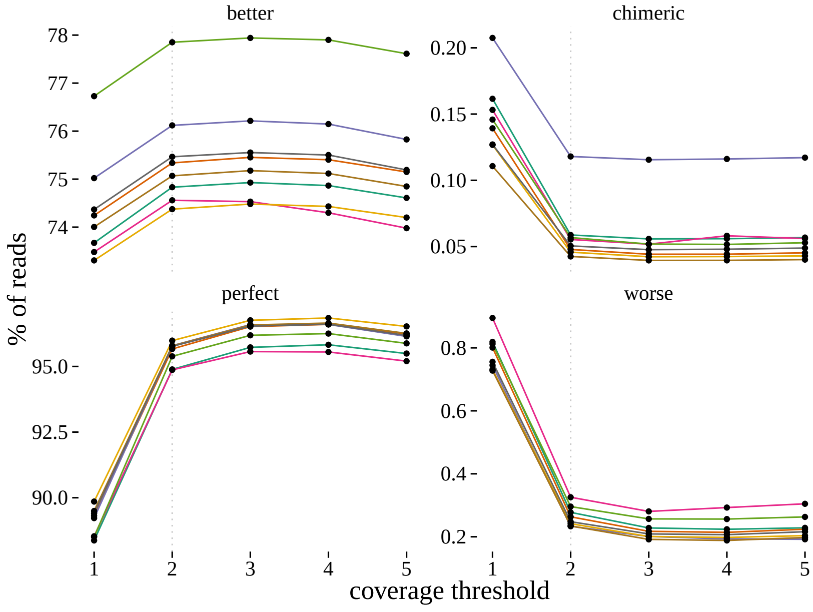

Figure S2: **Effect of different coverage thresholds.** MeCorS considers a *k*-mer trusted if it occurs at least twice in the metagenome.

By default, we consider a *k*-mer trusted if it occurs at least twice in the accompanying metagenome. The user can adjust this threshold this with the parameter `-c`, potentially improving MeCorS's performance for specific data sets.

Figure S2 shows the effect of a parameter sweep for the *E. coli* SAGs and the *in vitro* mock metagenome. Increasing the *k*-mer coverage threshold from 1 to 2 is the most beneficial, further increasing this threshold only marginally improves results. Above some coverage threshold error correction performance begins to decline, which we believe to be dependent on the taget genome's metagenomic coverage. For the *E. coli* SAGs and the concomitant mock metagenome, this turning point seems to be around 4.

k-mer size for error correction

The *k*-mer size does not depend on e.g. the read length, but on the *k*-mer's uniqueness in the (meta)genome. Kelley *et al.*, 2010[12], suggest a minimum *k*-mer size of 15 for bacterial genomes.

Following Li, 2015, MeCorS uses a larger *k*-mer size of 31 by default (and currently does not support larger *k*-mers).

[12] Kelley, D. R., Schatz, M. C., and Salzberg, S. L. (2010). Quake: quality-aware detection and correction of sequencing errors. *Genome Biol.*, **11**(11), R116

IDBA-UD assemblies

Metric	Program	SAG							
		0	1	2	3	4	6	7	8
NG50	raw	45284	53863	31250	24834	17196	87102	80574	59754
	BayesHammer	40924	51004	29256	24023	17246	**95532**	**87102**	44292
	MeCorS	**59081**	**73496**	**54946**	**41749**	**31569**	90184	80997	**80997**
# contigs	raw	330	342	514	607	654	201	200	303
	BayesHammer	335	345	530	626	653	**191**	**194**	328
	MeCorS	**277**	**272**	**409**	**497**	**512**	200	198	**249**
Largest contig	raw	227106	203026	**203098**	141383	102074	**221687**	**232585**	162612
	BayesHammer	203098	157125	197417	141494	107872	**221687**	178322	139398
	MeCorS	**236473**	**203098**	144213	**141579**	**124628**	221683	221683	**236473**
Total length	raw	4400079	4587934	4400469	4139052	3940625	4640153	**4639167**	4533515
	BayesHammer	4402128	**4591089**	4400334	4144742	3934141	**4641409**	4638005	**4538546**
	MeCorS	**4408926**	4590112	**4421966**	**4171137**	**3972787**	4639453	4636457	4538141
# misassemblies	raw	16	11	15	32	36	**0**	**0**	10
	BayesHammer	17	11	9	37	37	**0**	**0**	8
	MeCorS	**9**	**4**	**2**	**13**	**20**	**0**	**0**	**7**
# mismatches per 100 kbp	raw	**4.17**	**3.64**	10.16	17.02	20.41	0.31	0.13	3.16
	BayesHammer	4.88	3.88	12.50	16.35	20.76	**0.15**	**0.11**	**2.90**
	MeCorS	4.38	3.80	**7.93**	**8.86**	**12.82**	2.30	2.39	3.27
# indels per 100 kbp	raw	0.32	**0.24**	0.69	1.47	1.09	0.13	**0.09**	0.36
	BayesHammer	0.32	0.29	0.79	1.52	1.51	0.11	**0.09**	0.34
	MeCorS	**0.25**	0.31	**0.53**	**0.90**	**0.85**	**0.09**	**0.09**	**0.18**
Genome fraction (%)	raw	93.656	97.182	93.344	87.892	83.101	98.266	98.245	96.104
	BayesHammer	93.631	97.165	93.304	87.924	82.958	98.211	98.175	96.028
	MeCorS	**93.928**	**97.431**	**93.988**	**88.827**	**84.053**	**98.271**	**98.252**	**96.265**
# genes	raw	3873	4049	3741	3477	3220	4186	4191	4027
	BayesHammer	3859	4052	3709	3469	3208	4194	4199	4005
	MeCorS	**3931**	**4126**	**3857**	**3593**	**3347**	**4204**	**4202**	**4088**

Table S3: **IDBA-UD assembly results.** Quality assessment with QUAST.

We assembled all sets of reads with IDBA-UD[13], version 1.1.2. We modified its source code to increase the maximum allowed read length from 128 to 256 by modifying `kMaxShortSequence` in `src/sequence/short_sequence.h`, as described by the developers.

[13] Peng, Y. *et al.* (2012). IDBA-UD: a de novo assembler for single-cell and metagenomic sequencing data with highly uneven depth. *Bioinformatics*, **28**(11), 1420–1428

IDBA-UD expects read pairs interleaved and in Fasta format. For conversion we used IDBA-UD's helper tool `fq2fa`:

```
for f in Eco*.fastq.1.gz; do
	fq2fa --merge <(zcat $f) <(zcat ${f/.1./.2.}) ${f/1.gz/fasta}
done
```

Then, we assembled all read sets with `idba_ud` (default settings):

```
for f in Eco*.fasta; do
	echo idba_ud -r $f -o idba_${f%.fasta}
done
```

Lastly, we used QUAST[14], version 3.1, to evaluate the IDBA-UD assemblies.

[14] Gurevich, A. *et al.* (2013). QUAST: quality assessment tool for genome assemblies. *Bioinformatics*, **29**(8), 1072–1075

SPAdes assemblies

		SAG							
Metric	Program	0	1	2	3	4	6	7	8
NG50	raw	65444	95218	66287	48903	26823	114661	117715	86966
	BayesHammer	**86625**	95218	67436	52817	31448	**120770**	**132608**	105995
	MeCorS	**86625**	**95517**	**72055**	**53947**	**36236**	112350	**132608**	**112853**
# contigs	raw	447	400	606	718	813	245	233	324
	BayesHammer	302	**275**	474	594	676	**198**	**185**	**250**
	MeCorS	**288**	279	**418**	**534**	**569**	210	213	263
Largest contig	raw	203603	**224667**	**218793**	**178300**	113773	269308	268816	223154
	BayesHammer	**204882**	203257	**218793**	167410	135551	**312119**	269348	**269318**
	MeCorS	203394	224320	**218793**	178231	**155221**	268535	**312008**	268327
Total length	raw	**4522153**	**4703061**	**4533214**	**4290463**	**4138591**	**4713277**	**4718163**	**4633297**
	BayesHammer	4443696	4633876	4464269	4233011	4046113	4686582	4689565	4584513
	MeCorS	4452849	4645907	4471868	4240428	4065827	4698390	4702810	4600454
# misassemblies	raw	15	**2**	22	45	51	**1**	3	12
	BayesHammer	11	7	19	31	38	**1**	2	7
	MeCorS	**6**	3	**10**	**23**	**22**	**1**	**0**	**6**
# mismatches per 100 kbp	raw	15.30	11.57	34.70	48.21	50.53	2.84	**2.14**	9.72
	BayesHammer	12.70	10.30	30.34	40.41	48.42	**1.27**	2.17	**7.66**
	MeCorS	**10.41**	**9.32**	**22.69**	**30.86**	**36.47**	5.66	5.21	8.43
# indels per 100 kbp	raw	0.89	1.17	2.26	4.48	4.24	0.31	**0.22**	1.00
	BayesHammer	1.17	1.19	3.16	3.48	4.58	**0.24**	0.35	0.94
	MeCorS	**0.64**	**0.95**	**2.24**	**3.30**	**3.28**	0.55	0.31	**0.83**
Genome fraction (%)	raw	**94.241**	**97.948**	**94.239**	89.098	84.372	**98.665**	**98.708**	**96.702**
	BayesHammer	94.050	97.527	93.984	89.133	84.220	98.505	98.446	96.459
	MeCorS	94.223	97.629	94.223	**89.543**	**84.798**	98.580	98.541	96.603
# genes	raw	3876	4117	3782	3532	3281	4217	**4224**	4081
	BayesHammer	3898	4124	3805	3562	3300	4211	4219	4093
	MeCorS	**3937**	**4133**	**3866**	**3608**	**3390**	**4218**	4220	**4097**

Table S4: **SPAdes assembly results.** Quality assessment with QUAST.

We assembled all read sets with SPAdes, version 3.6.0:

```
for f in Eco*.fastq.1.gz; do
	spades.py --sc --only-assembler --careful -k 21,33,55,77 \
	-1 $f -2 ${f/.1./.2.} -o spades_${f%.fastq.1.gz}
done
```

The parameter `--careful` minimizes the number of mismatches in the final contigs. We empirically selected `-k 21,33,55,77` to account for longer SAG sequencing reads. Iterating over these four *k*-mer sizes generated assemblies of higher contiguity than the default settings of `-k 21,33,55`, while maintaining a high accuracy.

We used QUAST again to evaluate the SPAdes assemblies. While there are subtle differences between the IDBA-UD and SPAdes assemblies, both results demonstrate the large potential of metagenome-enabled error correction.

Running MeCorS multithreaded

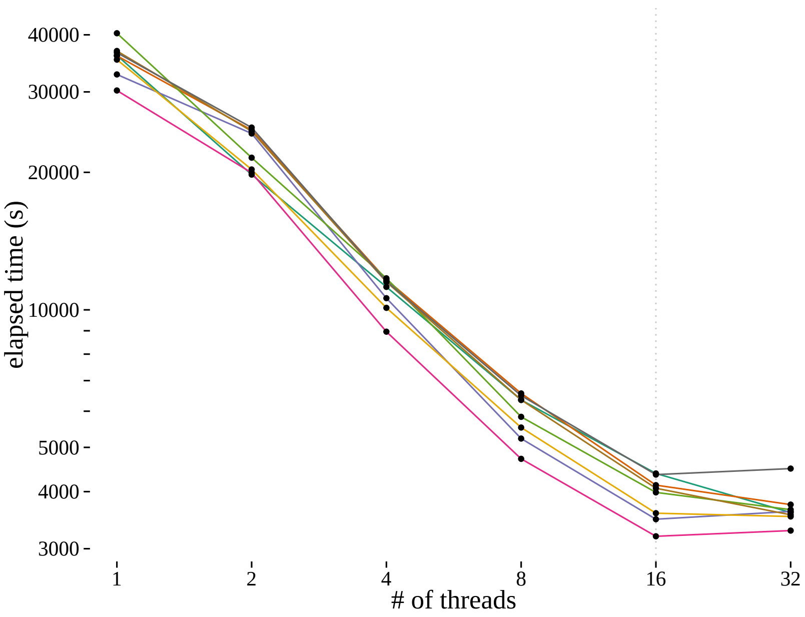

Figure S3: **Effeciency of threading.** MeCorS defaults to using 16 threads.

MeCorS uses the same multithreading strategy as e.g. bwa (and re-uses chunks of Heng Li's source code, released under the MIT license). Briefly, MeCorS first reads a batch of N sequencing reads containing B bases, and then processes these in t threads.

On our systems, we seem to run into an I/O bottleneck using 16 threads and a batch size of 100 Mbp. Increasing the batch size and/or the number of threads did not speed up computation, therefore we picked these as defaults. The parameters `-B` and `-t` allow the user to adjust these settings.

References

Bankevich, A. *et al.* (2012). SPAdes: a new genome assembly algorithm and its applications to single-cell sequencing. *J. Comput. Biol.*, **19**(5), 455–477.

Bowers, R. M. *et al.* (2015). Impact of library preparation protocols and template quantity on the metagenomic reconstruction of a mock microbial community. *BMC Genomics*, **16**(1), 856.

Clingenpeel, S. *et al.* (2014a). Effects of sample treatments on genome recovery via single-cell genomics. *ISME J*, **8**(12), 2546–2549.

Clingenpeel, S. *et al.* (2014b). Reconstructing each cell's genome within complex microbial communities–dream or reality? *Front Microbiol*, **5**, 771.

Gurevich, A. *et al.* (2013). QUAST: quality assessment tool for genome assemblies. *Bioinformatics*, **29**(8), 1072–1075.

Kelley, D. R., Schatz, M. C., and Salzberg, S. L. (2010). Quake: quality-aware detection and correction of sequencing errors. *Genome Biol.*, **11**(11), R116.

Lasken, R. S. and Stockwell, T. B. (2007). Mechanism of chimera formation during the Multiple Displacement Amplification reaction. *BMC Biotechnol.*, **7**, 19.

Li, H. (2013). Aligning sequence reads, clone sequences and assembly contigs with BWA-MEM. *arXiv:1303.3997*.

Li, H. (2015). BFC: correcting Illumina sequencing errors. *Bioinformatics*, **31**(17), 2885–2887.

Li, H. and Durbin, R. (2010). Fast and accurate long-read alignment with Burrows-Wheeler transform. *Bioinformatics*, **26**(5), 589–595.

Li, H. *et al.* (2009). The Sequence Alignment/Map format and SAMtools. *Bioinformatics*, **25**(16), 2078–2079.

Nikolenko, S. I. *et al.* (2013). BayesHammer: Bayesian clustering for error correction in single-cell sequencing. *BMC Genomics*, **14 Suppl 1**, S7.

Peng, Y. *et al.* (2012). IDBA-UD: a de novo assembler for single-cell and metagenomic sequencing data with highly uneven depth. *Bioinformatics*, **28**(11), 1420–1428.
